# Supplementary material for: Illumina TruSeq Synthetic Long-Reads Empower De Novo Assembly and Resolve Complex, Highly-Repetitive Transposable Elements
Source: PLoS One. 2014 Sep 4;9(9):e106689. doi: 10.1371/journal.pone.0106689 (PMC4154752; doi:10.1371/journal.pone.0106689)
Supplement: File S1 — Supplemental materials describing the generation of synthetic long-reads from underlying short-read data, supplemental figures, and supplemental tables. (PDF) [file pone.0106689.s001.pdf]

Illumina TruSeq synthetic long-reads empower *de novo* assembly and resolve complex, highly-repetitive transposable elements

Rajiv C. McCoy[1], Ryan W. Taylor[1], Timothy A. Blauwkamp[2], Joanna L. Kelley[3], Michael Kertesz[4], Dmitry Pushkarev[5], Dmitri A. Petrov*[1] and Anna-Sophie Fiston-Lavier*[1,6]

[1]Department of Biology, Stanford University, Stanford, California 94305, USA
[2]Illumina Inc., Hayward, California 94545, USA
[3]School of Biological Sciences, Washington State University, Pullman, Washington 99164, USA
[4]Department of Bioengineering, Stanford University, Stanford, California 94035, USA
[5]Department of Physics, Stanford University, Stanford, California 94035, USA
[6]Institut des Sciences de l'Evolution-Montpellier, Montpellier, Cedex 5, France
Corresponding author: Rajiv C. McCoy rmccoy@stanford.edu
*DAP and ASFL are joint senior authors on this work.

Supplemental Materials

Generation of TruSeq™ synthetic long-reads from short read data

Short Read Pre-Processing

Prior to the assembly of the synthetic long-reads, the short reads in every well are pre-filtered to correct for errors which could lead to mis-assemblies. Reads that do not have a sufficient stretch of high-quality bases are filtered. Low-quality ends of remaining bases are trimmed (hard-clipped). Read pairs that appear to 'read through' one another, and thus potentially contain adapter sequence on the 3' end(s) of one or both reads, are modified as follows. The first read is trimmed of bases that appear to extend beyond the second read, and the second read is discarded, resulting in an unpaired read that should have had any 3' adapter sequence clipped off. If the trimmed reads in a pair are shorter than 30 bp, the pair is discarded. If one read in a pair is shorter than 30 bp, and the second read longer than 50 bp, the longer read is kept. Adapter sequences are removed and the end-marker sequences identified and trimmed, and reads containing end-marker sequences are tagged for downstream use in the pipeline.

Assembly of Contigs

The assembly module consists of several steps: digital normalization, read error correction, graph construction, and clean-up using paired-end reads. These steps are described in more detail in the following sections.

Digital Normalization

Due to bias introduced during PCR, the read coverage among input fragments in the sample can vary greatly. In order to normalize coverage variation across fragments (which improves the accuracy of the assembly as well

as the computational performance of the algorithm), digital normalization methods are used [1]. The digital normalization process smooths out highly-biased sequence coverage by removing specific over-represented sequences. Coverage is normalized such that the highest-coverage fragments are approximately 40×.

Error Correction

Following digital normalization, an error correction step is performed using an overlap-based method. The aim of this step is to correct PCR and sequencing artifacts which introduce false base substitutions or indels. At a high level, it operates as follows. An index of all k-mers of length 31 in the reads is constructed (the k-mer hash). For each read, k-mers in the read are compared to the index to find the set of reads which share the same k-mer. Matches to candidate overlapping reads are extended using semi-banded global alignment, and those which have a match length of at least 31 bases and share 95% identity are retained. Multiple sequence alignment (MSA) of the set of overlapping reads is performed. Using both the base quality scores of the reads and the results of the MSA, a consensus sequence for the read is generated.

Graph Construction

The main assembly step is performed using the String Graph Assembler (SGA) [2], which is an overlap-based assembly method. In the first stage, SGA uses a k-mer overlap size of 31 to create a graph with reads as vertices and k-mer overlaps as edges.

After the construction of an initial graph, the next step of the algorithm is to clean the graph and remove spurious edges using several heuristics. The algorithm requires that paths in the graph are supported by paired-end reads. It checks for the existence of a path linking the two reads of a read pair within the expected insert size distribution (500 bp, by default). Any edges in the graph which do not support read pairs are removed. In addition, tips and bubbles in the read graph, which normally occur during *de novo* assembly, are cleaned up using standard graph-cleaning methods.

Scaffolding Contigs to Assemble Long Reads

The next stage in the pipeline is scaffolding, the goal of which is to use paired-end information to place and orient the contigs generated in the previous step and fill in gaps between contigs. The method employed in the long reads pipeline is based on the scaffolding method used in the original SGA assembler, and the user is referred to the original paper for further details [2].

In brief, scaffolding is accomplished by re-aligning the input short reads to the contigs using BWA aligner

[3], and using the paired-end alignments to infer scaffold structure. The link between two contigs is made when two or more paired reads map such that read 1 from a read pair maps to one contig and read 2 from the same read pair maps to the other. The orientation of the contigs relative to one another is also inferred from the orientation of the read pairs. In addition, the end-marker sequences are used to help guide and constrain the construction of our scaffold graph

Gap Filling

The next step of this module is to fill in scaffold gaps where possible in order to resolve repeats. In this step, we use the input short reads, making use of the FM index computed during the contig assembly. We begin by finding the highest-scoring read which matches the end of one of the contigs, and continue to chain together reads iteratively. If a chain is found that overlaps another contig in the same scaffold, the consensus is retained and the gap filled with this sequence.

Assembly QC and Correction

The final stage of the analysis pipeline involves verification of the scaffolds and error correction. The short read data is again aligned against the scaffolds generated in the previous step using BWA aligner [3]. Based on the alignments, the scaffolds are corrected for single-nucleotide errors and broken into smaller scaffolds should there be only partial alignment support. Quality scores for the final long reads are also estimated from the alignments.

Breaking Scaffolds

The short reads used during the synthetic long-read assembly are aligned to the scaffolds. The alignments are searched for read pairs in which one read aligns and the other one does not. Unaligned reads are re-aligned, and reads that are overlapping or running into scaffold gaps are counted and computed. In order to determine whether or not to break a scaffold gap, Illumina computes the following formula:

```
   sqrt(0.3+(reads aligning to mid point of gap on fwd strand)*(0.3+
(reads aligning to mid point of gap on rev strand)))/(total
      number of reads in gap)
```

If this ratio is smaller than 0.1, the gap is left as is. If it is larger, the scaffold is broken at this gap. If there are only few reads or none, the scaffold for the region is left as is.

Q-scores

From the alignments of short reads to the scaffolds, a pileup file is generated which provides the base quality scores of the aligned reads at each position in a scaffold. The quality score at each scaffold position is then estimated from the read base qualities as follows:

- Remove 'N's and indels from the pileup.
- If coverage $> 5\times$ and all nucleotides at this position agree, set Q-score to max of pileup.
- If $< 5\%$ mismatches or > 3 matches, set Q-score to mean of pileup.
- If all of the above steps fail, look at the most frequently-occurring nucleotide in the pileup as well as the second most frequent nucleotide. Compute the posterior probability of most frequent base given the quality scores. This includes some correction factors from a PCR error rate model. Do the same for the second most frequent nucleotide. Choose the nucleotide with the highest posterior probability and compute the Q-score from this probability.

Pre-assembly quality control

Assessment of contamination

We assessed the degree of contamination with BLASTN [4] by searching against the NCBI nucleotide database (see Methods). The degree of contamination in the TruSeq synthetic long-read libraries was low, with 99.8% (953,797) of reads having top hits to *D. melanogaster* reference sequences. We note that the number of synthetic long-reads with top BLASTN hits to *D. melanogaster* is lower than the number that map to the reference genome with BWA-MEM for several reasons. First, a small number of reads derived from regions of extremely low divergence erroneously map to other *Drosophila* species. Second, the "Uextra" scaffolds likely contain some contamination from other species as described in the release notes: `http://www.fruitfly.org/data/sequence/README.RELEASE5`. Finally, for a very small number of reads, large proportions of the reads lengths are clipped by BWA-MEM with only small subsequences that align. Based on the BLASTN results, the most abundant contaminant reads had top matches to known symbionts of *D. melanogaster*, including acetic acid bacteria from the genera *Gluconacetobacter*, *Gluconobacter*, and *Acetobacter* (Table S2 in Supporting File S1). Because contamination was extremely rare and because we could not exclude that sequences with no BLAST hits may correspond to fly-derived sequences not previously assembled in the reference genome, we included all sequences in downstream analyses.

Genome assembly from TruSeq synthetic long-reads

Assembly with the Celera Assembler

The following Celera Assembler parameters are roughly based on those recommended for PacBio consensus-corrected reads: `http://sourceforge.net/apps/mediawiki/wgs-assembler/index.php?title=PBcR#Assembly_of_Corrected_Sequences`. Based on our goal of assembling separate copies of TEs, however, we elected to use a greater k-mer size and k-mer threshold to increase specificity and reduce the number of false joins (which could generate chimeric sequences).

```
unitigger=bogart
merSize=31
merThreshold=auto*2
ovlMinLen=800
obtErrorRate=0.03
obtErrorLimit=4.5
ovlErrorRate=0.03
utgErrorRate=0.015
utgGraphErrorRate=0.015
utgGraphErrorLimit=0
utgMergeErrorRate=0.03
utgMergeErrorLimit=0
```

The bogart unitigger, which is recommended for Illumina data or Illumina data in combination with other data types, and is also employed in the PacBio corrected read assembly pipeline. We required overlap of at least 800 bp in order to merge across reads, a parameter that further increases overlap specificity. Error rates are set substantially lower than the default options, given the low observed rate of mismatches to the reference genome in the TruSeq synthetic long reads as well as the fact that we sequenced a highly inbred strain of *D. melanogaster*. These parameters are intentionally conservative to avoid the erroneous merging of contigs at identical repeats. Modifications to these parameters may increase overlap sensitivity and achieve greater contig lengths, but likely at the expense of mis-assembly. Assembly for species with higher rates of polymorphism would require error rates to be set higher to avoid separate assembly of individual haplotypes.

Contig merging with Minimus2

NUCmer [5, 6] alignment to the reference genome revealed that in some cases, the Celera Assembler produced contigs with ends with long stretches (>1 Kbp) of perfect sequence identity. As we demonstrated in the main text, many of these cases represent regions of low coverage in synthetic long reads, where data were insufficient to support a join. We therefore used the simple overlap-based assembler Minimus2 to generate supercontigs from the contigs output by Celera. The parameters used for this assembly were:

```
REFCOUNT= 0
MINID   = 99.9
OVERLAP = 800
MAXTRIM = 1000
WIGGLE  = 15
CONSERR = 0.01
```

The parameter REFCOUNT=0 means that the assembler performs all vs. all alignment of the contigs, rather than merging two separate assemblies (a common application of Minimus2). We required a stringent sequence identity of 99.9% with at least 800 bp of overlap at the contig ends to allow a join, thereby avoiding false contig joins.

Assembly assessment with NUCmer alignment

Alignment of assembled contigs to the high quality reference genome was performed with NUCmer (version 3.23) [5, 6], and the resulting alignment file was filtered according to guidelines described in the documentation: `http://mummer.sourceforge.net/manual/#mappingdraft`.

```
nucmer ref.fasta qry.fasta

delta-filter -q out.delta > out.q.delta
```

We required alignments to have at least 99% identity to the reference for at least 1000 bp.

```
show-coords -THrcl out.q.delta | \
     awk '{if ($7>99 && $5>1000) print $12"\t"$1"\t"$2"\t"$13"\t"$11}' > nucmer.bed
```

We then used BEDTools (version 2.19.1) [7] to merge across perfectly adjacent or partially overlapping alignments.

```
bedtools merge -i nucmer.bed > nucmer.merge.bed
```

Alignment statistics reported in Table 2 were then produced as follows:

```
for i in X 2L 2R 3L 3R 4 XHet 2LHet 2RHet 3LHet 3RHet YHet M U
do
     echo $i
     # count the alignments
     cat nucmer.bed | awk -v i=$i '{if ($1==i) print}' | cut -f4 | sort | uniq | wc -l

     # count the gaps
 bedtools complement -g reference.genome -i nucmer.merge.bed > nucmer.complement.bed
 cat nucmer.complement.bed | awk -v i=$i '{if ($1==i) print}' | wc -l

     # sum the total aligned length
     cat nucmer.merge.bed | awk -v i=$i '{if ($1==i) print $3-$2}' | \
          awk '{sum+=$1} END {print sum}'
     printf "\n\n"
done
```

The same alignment file (.delta) is also analyzed to define the search space for TEs and genes: `https://github.com/rmccoy7541/assess-assembly`. The steps in the pipeline are as follows:

- Map contigs to the reference genome with NUCmer, extracting only the optimal mapping of each contig to one position in the reference.
- Check whether both the start and end boundary of the gene or TE fall within the same aligned contig.
- If so, perform local alignment between the reference sequence of the gene or TE and the corresponding aligned sequence.
- Calculate the percent identity and the proportion of the gene or TE's length that was assembled and aligned.

Supplemental Figures

Figure 1: Diagram of the TruSeq synthetic long-read library preparation protocol.

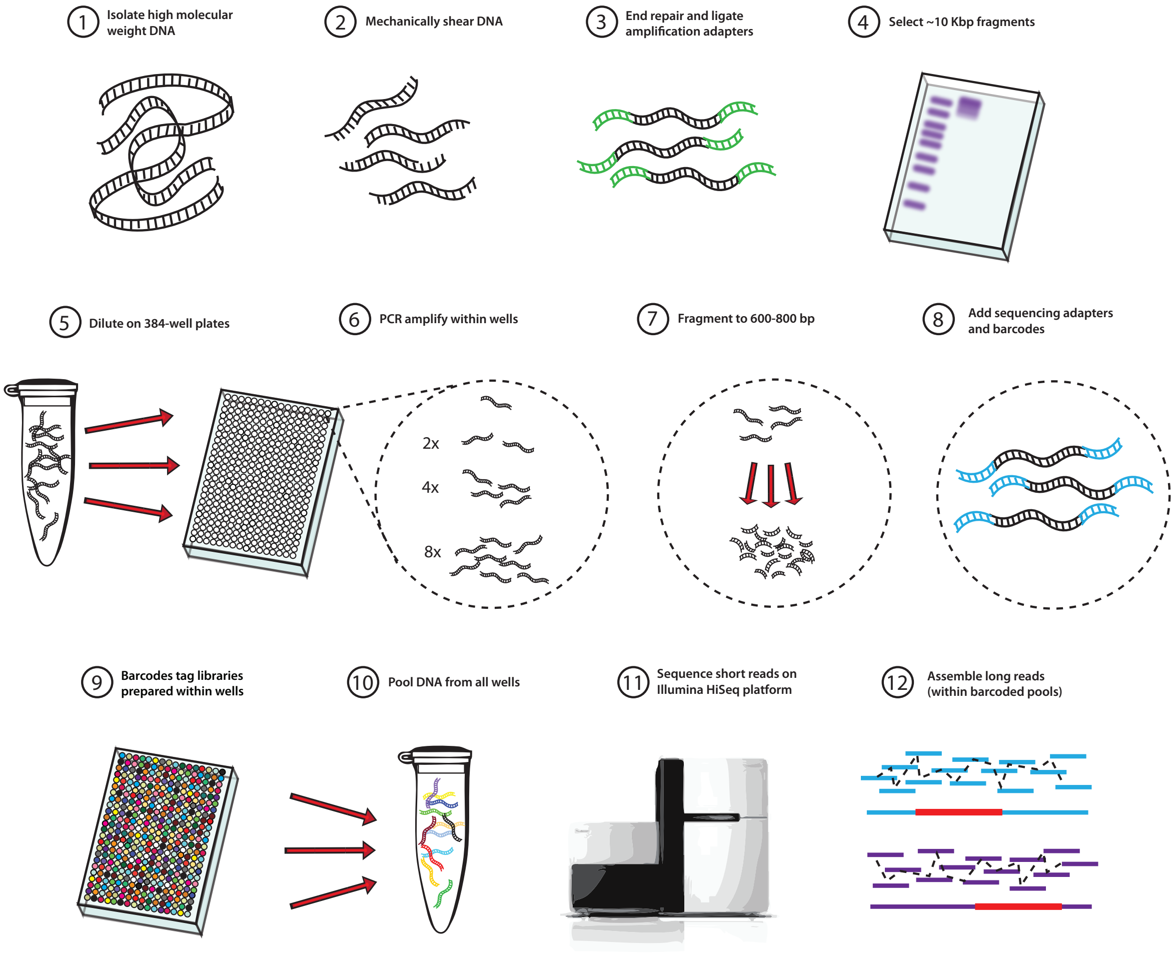

Figure 2: Dot plots depicting NUCmer [5] alignment between assembled contigs and the reference genome. Segments off of the diagonal represent various classes of mis-assembly (insertions, deletions, or translocations with respect to the reference sequence). Red segments represent forward alignments, while blue segments indicate an inversion with respect to the rest of the contig alignment. Dot plots were generated using the mummerplot feature of MUMmer [6]

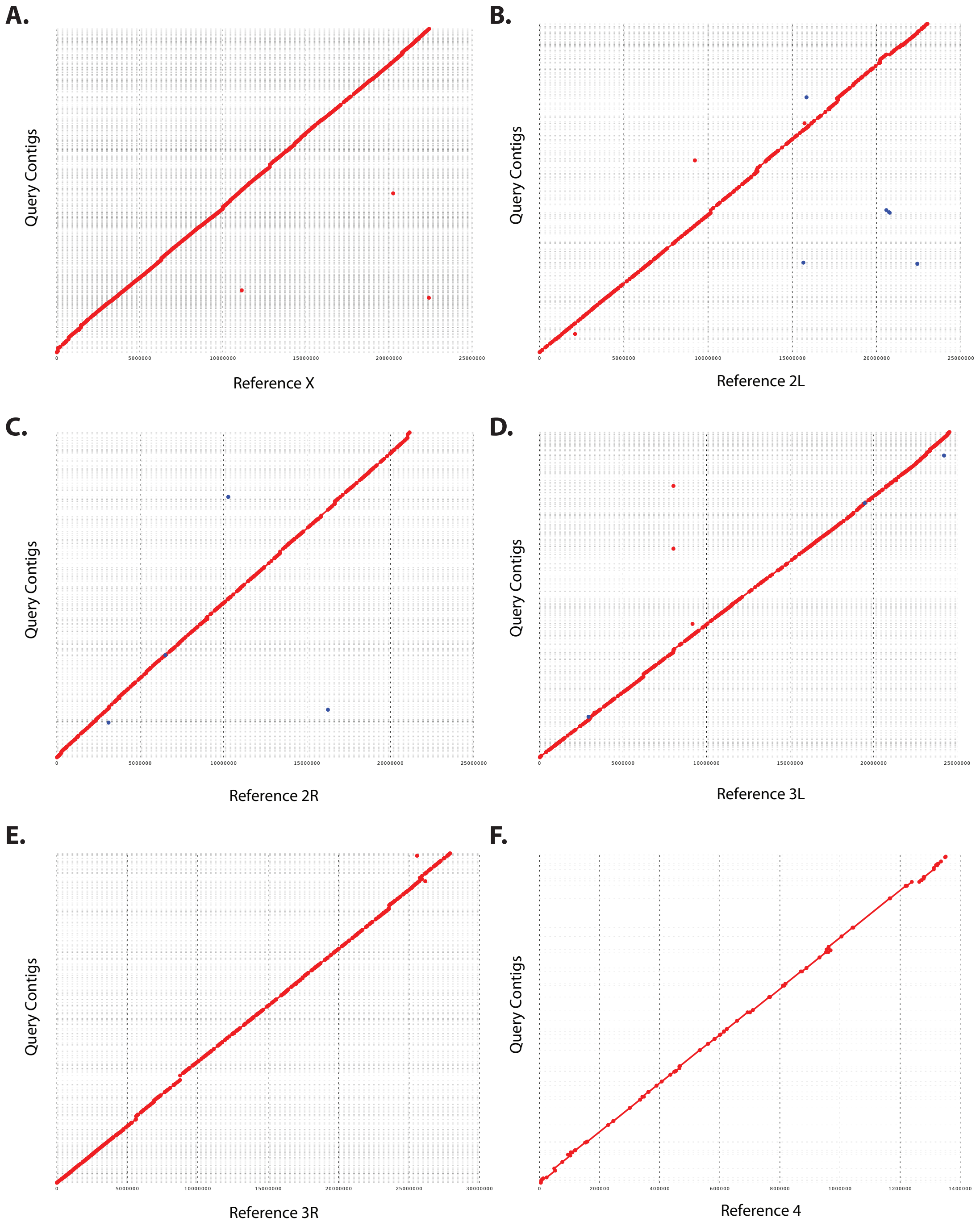

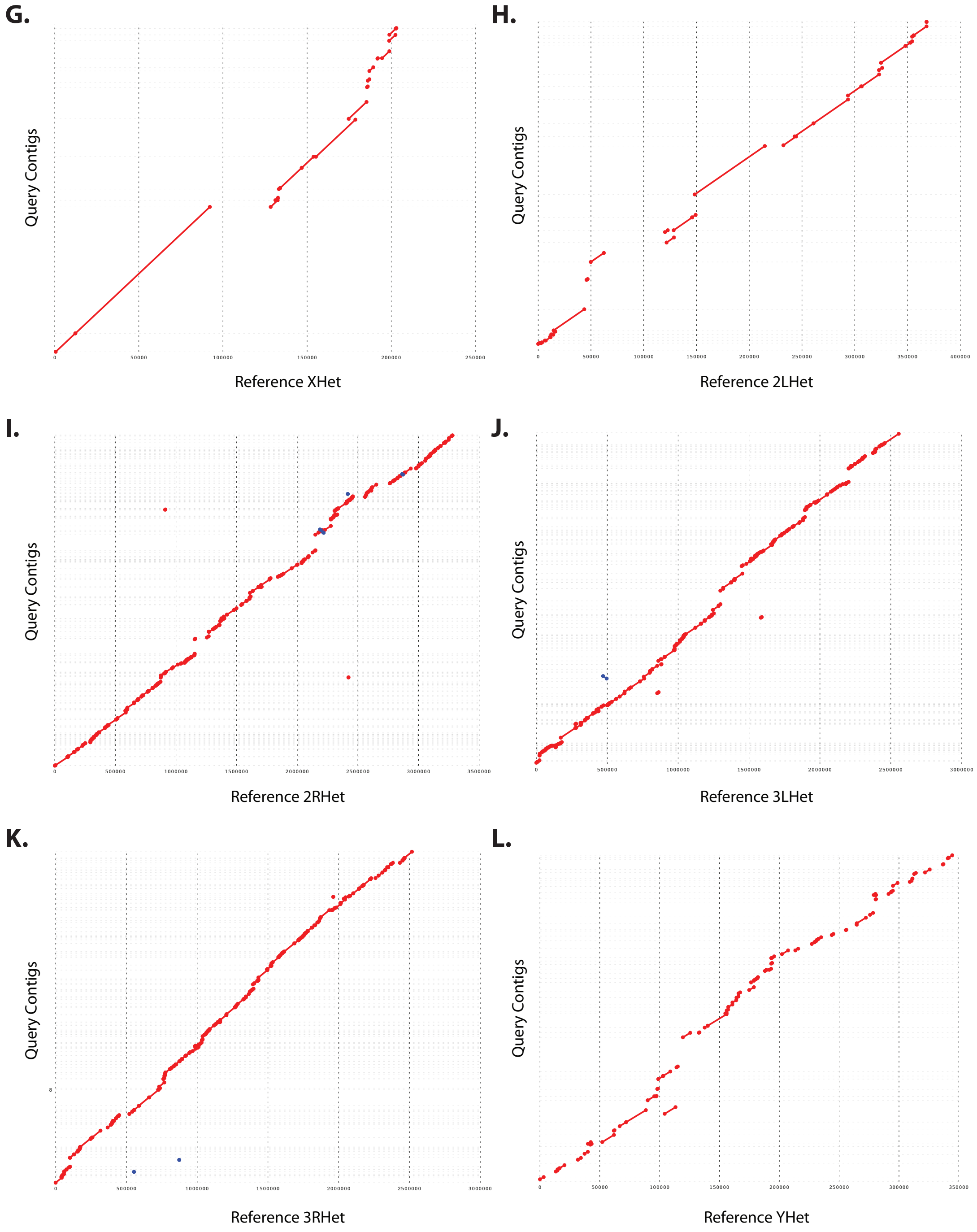
G.
Query Contigs
Reference XHet
H.
Query Contigs
Reference 2LHet
I.
Query Contigs
Reference 2RHet
J.
Query Contigs
Reference 3LHet
K.
Query Contigs
Reference 3RHet
L.
Query Contigs
Reference YHet

Figure 3: IGV screenshot [8] of a representative case where assembly fails due to a deficiency of long-read data derived from a long transposable element sequence. The upper-most track (blue) represents the NUCmer alignment of assembled contigs to the reference genome. The middle track represents the BWA alignment of the underlying TruSeq synthetic long-reads. For each of these tracks, blue and red shading indicate the orientation of the alignment (i.e. whether the sequence is reverse complemented). The bottom tracks (blue) indicates the boundaries of genes and transposable elements.

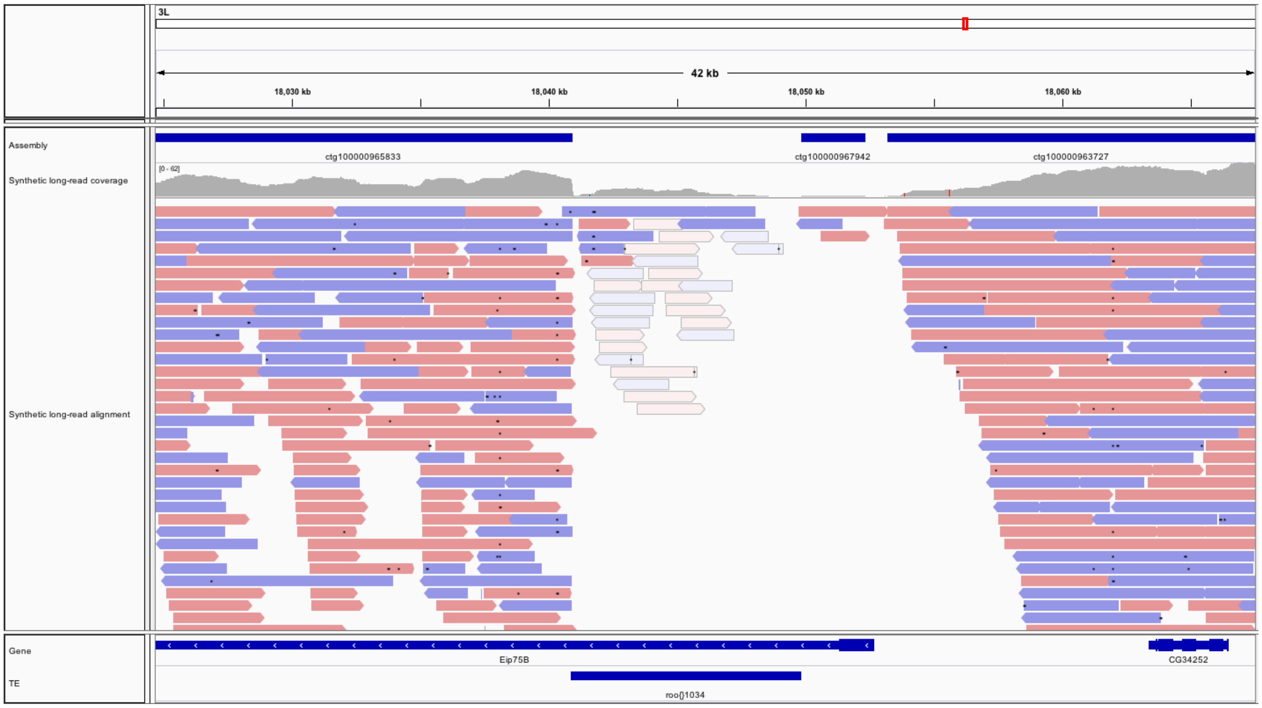

Figure 4: IGV screenshots [8] of representative cases where assembly succeeds or fails based on characteristics of TEs in the genomic region. See the legend of Figure S4 for descriptions of each of the alignment tracks. **A:** A case where assembly fails in the presence of tandem repeats of elements from the Dm88 family. **B:** A case where assembly succeeds in a repeat-dense region of chromosome arm 2R.

A.

B.

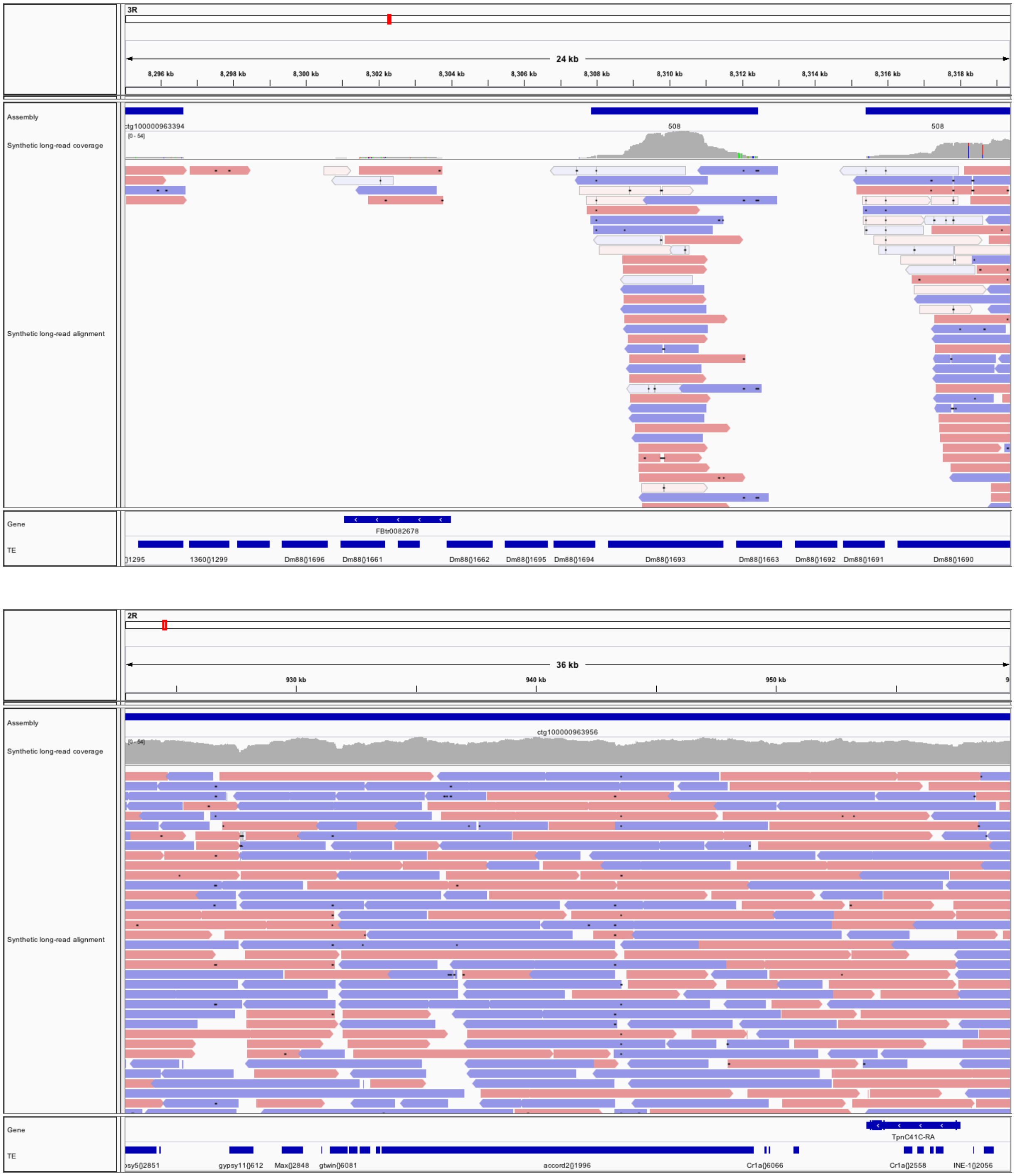

Supplemental Tables

Table S1: Number of read pairs in Illumina short read libraries (2×100 bp) and corresponding TruSeq synthetic long-read libraries (1.5-15 Kbp). In the case of mol-32-2827 and mol-32-283d, short read data from separate flow cells were combined, as indicated.

Short read library ID	Flow cell & lane ID	No. read pairs	TruSeq library ID	No. synthetic long-reads
LP6005512-DNA_A01-LRAAA-05	D2672ACXX, 1	212463575	mol-32-281c	170951
LP6005512-DNA_A01-LRAAA-06	D2672ACXX, 2	203972521	mol-32-2827	240750
	D2B7LACXX, 7	82066168		
LP6005512-DNA_A01-LRAAA-07	D2672ACXX, 3	196599647	mol-32-2832	174387
LP6005512-DNA_A01-LRAAA-08	D2672ACXX, 4	154537575	mol-32-283d	254770
	D2B7LACXX, 8	175910619		
LP6005512-DNA_A01-LRAAA-09	C2A96ACXX, 3	174398573	mol-32-2f5f	59705
LP6005512-DNA_A01-LRAAA-10	C2A96ACXX, 4	182493763	mol-32-2f6a	55273

Table S2: Top BLAST hits to the NCBI nucleotide database for all TruSeq synthetic long-reads. Only species/strains with $\geq$6 hits are reported here.

No. long reads	Species/strain of top BLAST hit
953797	*Drosophila melanogaster*
214	*Gluconacetobacter diazotrophicus* PAl 5
175	*Enterobacteria* phage HK629
163	*Gluconacetobacter xylinus* E25
114	*Gluconacetobacter xylinus* NBRC 3288
97	*Gluconobacter oxydans* 621H
96	*Drosophila mauritiana*
83	*Gluconobacter oxydans* H24
76	*Acetobacter pasteurianus* 386B
58	Cloning vector pSport1
44	*Drosophila pseudoobscura pseudoobscura*
30	*Drosophila simulans*
30	synthetic construct
25	*Acetobacter pasteurianus* IFO 3283-01
14	*Drosophila sechellia*
10	*Burkholderia lata*
9	*Cloning vector* placZ.attB
8	*Acetobacter aceti* NBRC 14818
7	*Acetobacter pasteurianus* IFO 3283-01/12
7	*Agrobacterium fabrum* str. C58
7	*Azospirillum brasilense* Sp245
6	*Granulibacter bethesdensis* CGDNIH3
6	*Rhodomicrobium vannielii* ATCC 17100
6	*Zymomonas mobilis mobilis* ATCC 29191

Table S3: Family membership of TEs overlapping gaps in the alignment of the genome assembly to the high quality reference genome. Families with $\geq$10 overlaps are reported here.

Family	No. TE copies
roo	117
INE-1	84
1360	34
F	26
FB	21
invader4	20
297	18
mdg1	16
Dm88	15
Doc	15
Tirant	14
HMS-Beagle	11
opus	11
copia	10
invader1	10
invader3	10

Table S4: Assembly results for all annotated transposable elements in the *D. melanogaster* genome. As in Kaminker et al. [9], we report the average length of TE copies within each family, the average divergence between each copy and the canonical sequence, and the number of elements that comprise each family. We then report the number of elements of each family entirely recovered in our assembly with perfect identity to the reference genome, as well as the number that are partially recovered, mis-assembled, or contain mismatches relative to the reference. Finally, we report the number of elements from each family that are entirely absent from the assembly (i.e., both start and end coordinates lie within alignment gaps).

Family	Length	Divergence	Total	Full length	Partial/Mis-assembled	Absent
1360	758	0.059	304	241	56	7
17.6	4852	0.014	20	6	14	0
1731	1112	0.109	13	10	3	0
297	3906	0.044	80	35	41	4
3S18	2816	0.070	17	11	2	4
412	5414	0.036	37	11	25	1
accord	1976	0.195	3	2	1	0
accord2	3707	0.089	7	6	1	0
aurora	3124	NA	1	1	0	0
baggins	1625	0.027	35	29	4	2
Bari1	1447	0.019	6	6	0	0
Bari2	663	0.103	5	5	0	0
blood	7121	0.008	25	1	24	0
BS	1074	0.040	43	37	6	0
BS3	703	0.037	29	28	0	1
BS4	749	NA	1	1	0	0
Burdock	3319	0.050	22	10	12	0
Circe	2473	0.122	5	4	1	0
copia	4233	0.020	35	6	29	0
Cr1a	1597	0.092	152	136	14	2
diver	5029	0.039	11	1	9	1
diver2	1231	0.107	47	39	5	3
Dm88	1698	0.144	31	9	10	12
Doc	3386	0.025	68	19	41	8
Doc2	1688	0.161	7	5	2	0
Doc3	1229	0.259	21	17	3	1
Doc4	1925	0.315	7	7	0	0
F	3025	0.108	70	30	39	1
FB	1063	0.129	60	37	21	2
flea	3358	0.077	29	11	17	1
frogger	1986	NA	2	1	1	0
Fw2	1683	0.196	9	8	1	0
Fw3	423	NA	7	6	1	0
G	916	0.227	17	12	5	0
G2	1051	0.067	22	20	2	0
G3	1996	0.095	7	6	1	0
G4	1212	0.038	28	27	1	0
G5	994	0.069	25	22	3	0
G5A	735	0.063	27	27	0	0
G6	1346	0.112	10	10	0	0
G7	553	0.048	4	4	0	0
GATE	2915	0.080	20	11	7	2

Table S4 continued: Assembly results for all annotated transposable elements in the *D. melanogaster* genome. As in Kaminker et al. [9], we report the average length of TE copies within each family, the average divergence between each copy and the canonical sequence, and the number of elements that comprise each family. We then report the number of elements of each family entirely recovered in our assembly with perfect identity to the reference genome, as well as the number that are partially recovered, mis-assembled, or contain mismatches relative to the reference. Finally, we report the number of elements from each family that are entirely absent from the assembly (i.e., both start and end coordinates lie within alignment gaps).

Family	Length	Divergence	Total	Full length	Partial/Mis-assembled	Absent
gtwin	1559	0.084	19	17	1	1
gypsy	1514	0.147	18	17	0	1
gypsy2	2840	0.077	12	10	2	0
gypsy3	1629	0.126	15	13	2	0
gypsy4	1253	0.144	15	13	2	0
gypsy5	1879	0.144	10	7	3	0
gypsy6	1353	0.071	15	13	1	1
gypsy7	1292	0.126	4	4	0	0
gypsy8	980	0.103	57	54	1	2
gypsy9	1276	0.136	10	9	1	0
gypsy10	2886	0.086	7	7	0	0
gypsy11	1316	0.185	5	5	0	0
gypsy12	1391	0.103	50	45	4	1
H	1049	0.170	59	44	9	6
HB	1017	0.061	60	51	9	0
Helena	674	0.079	9	9	0	0
HeT-A	2436	0.036	25	8	17	0
HeT-Tag	21	0.012	23	1	22	0
HMS-Beagle	4610	0.043	23	7	14	2
HMS-Beagle2	2710	0.096	13	8	4	1
hopper	857	0.027	24	15	8	1
hopper2	1011	0.063	14	11	3	0
I	2350	0.113	38	24	8	6
Idefix	2169	0.114	17	12	5	0
INE-1	246	0.112	2235	2106	65	64
invader1	911	0.060	45	25	11	9
invader2	2196	0.063	19	12	6	1
invader3	1994	0.054	33	15	12	6
invader4	730	0.020	32	13	6	13
invader5	4175	0.106	3	2	1	0
invader6	1320	0.090	8	8	0	0
Ivk	2755	0.094	11	8	3	0
jockey	1605	0.040	96	76	16	4
jockey2	549	0.060	28	27	1	0
Juan	3272	0.037	11	9	2	0
looper1	1214	0.066	4	4	0	0
mariner2	627	0.064	23	22	1	0
Max	2393	0.302	21	17	4	0
McClintock	1781	0.046	8	5	2	1
mdg1	4894	0.052	41	12	25	4
mdg3	3254	0.034	21	9	10	2

Table S4 continued: Assembly results for all annotated transposable elements in the *D. melanogaster* genome. As in Kaminker et al. [9], we report the average length of TE copies within each family, the average divergence between each copy and the canonical sequence, and the number of elements that comprise each family. We then report the number of elements of each family entirely recovered in our assembly with perfect identity to the reference genome, as well as the number that are partially recovered, mis-assembled, or contain mismatches relative to the reference. Finally, we report the number of elements from each family that are entirely absent from the assembly (i.e., both start and end coordinates lie within alignment gaps).

Family	Length	Divergence	Total	Full length	Partial/Mis-assembled	Absent
micropia	1771	0.133	13	8	4	1
ninja-Dsim-like	1390	0.315	19	15	1	3
NOF	2609	0.071	8	2	4	2
opus	4824	0.074	31	9	21	1
pogo	651	0.006	48	44	4	0
Porto1	1090	0.013	7	7	0	0
Q	124	0.277	5	5	0	0
Quasimodo	3922	0.089	29	16	12	1
R1-2	802	NA	2	2	0	0
R1A1	1169	0.256	27	18	8	1
roo	7411	0.009	136	12	111	13
rooA	3654	0.053	17	12	5	0
rover	4091	0.041	7	4	3	0
Rt1a	2132	0.048	26	23	2	1
Rt1b	2945	0.046	60	45	12	3
Rt1c	1050	0.084	34	24	7	3
S	1102	0.471	65	48	16	1
S2	575	0.054	14	10	1	3
springer	2836	0.067	24	16	7	1
Stalker	2748	0.025	18	9	8	1
Stalker2	5853	0.043	16	7	9	0
Stalker3	31	NA	1	1	0	0
Stalker4	2559	0.054	37	22	12	3
Tabor	2330	0.059	9	6	3	0
TART-A	2928	0.038	11	5	2	4
TART-B	258	NA	3	2	1	0
TART-C	987	NA	1	1	0	0
Tc1	947	0.039	26	25	1	0
Tc1-2	857	0.049	24	23	1	0
Tc3	447	0.096	19	17	2	0
Tirant	6401	0.084	25	4	18	3
Tom1	292	0.055	4	4	0	0
transib1	4581	0.075	3	1	2	0
transib2	918	0.029	24	19	4	1
transib3	1493	0.027	13	11	2	0
transib4	1946	0.049	8	7	1	0
Transpac	4394	0.038	6	1	5	0
X	1466	0.233	55	50	4	1
Xanthias	4533	NA	1	0	1	0
Y	NA	NA	4	1	3	0
ZAM	547	0.508	4	4	0	0

Table S5: Results of fitting a generalized linear mixed model with a binary response variable indicating whether individual TE copies are accurately assembled.

Random effect		Variance	Std. Dev.	
Family	(Intercept)	1.330	1.153	

Fixed effect	Estimate	Std. Error	z value	$Pr(> \|z\|)$
(Intercept)	1.216	0.170	7.135	9.70×10^{-13}
Length	-1.633	0.079	-20.766	$< 2 \times 10^{-16}$
GC content	0.186	0.059	3.171	0.001 52
Divergence	0.692	0.092	7.501	6.35×10^{-14}
High identity copies	-0.529	0.180	-2.936	0.003 33
Divergence × High identity copies	0.382	0.097	3.921	8.81×10^{-5}

Table S6: Contig IDs for sequences with no significant hit to the NCBI nucleotide database.

FASTA contig ID
ctg100000966696
ctg100000966814
ctg100000966837
ctg100000967379
ctg100000967449
ctg100000967457
ctg100000967511
ctg100000967560
ctg100000967605
ctg100000967626
ctg100000967687
ctg100000967750
ctg100000967783
ctg100000967784
ctg100000967787
ctg100000967852
ctg100000967896
ctg100000967928
ctg100000967969
ctg100000968010
ctg100000968064
ctg100000968094
ctg100000968196
ctg100000968200
ctg100000968250
ctg100000968272
ctg100000968281

References

[1] Brown CT, Howe A, Zhang Q, Pyrkosz AB, Brom TH (2012) A reference-free algorithm for computational normalization of shotgun sequencing data. arXiv preprint arXiv:12034802 .

[2] Simpson JT, Durbin R (2012) Efficient *de novo* assembly of large genomes using compressed data structures. Genome Research 22: 549–556.

[3] Li H, Durbin R (2009) Fast and accurate short read alignment with Burrows-Wheeler transform. Bioinformatics 25: 1754–1760.

[4] Altschul SF, Madden TL, Schäffer AA, Zhang J, Zhang Z, et al. (1997) Gapped BLAST and PSI-BLAST: A new generation of protein database search programs. Nucleic Acids Research 25: 3389–3402.

[5] Delcher AL, Phillippy A, Carlton J, Salzberg SL (2002) Fast algorithms for large-scale genome alignment and comparison. Nucleic Acids Research 30: 2478–2483.

[6] Kurtz S, Phillippy A, Delcher AL, Smoot M, Shumway M, et al. (2004) Versatile and open software for comparing large genomes. Genome Biology 5: R12.

[7] Quinlan AR, Hall IM (2010) BEDTools: A flexible suite of utilities for comparing genomic features. Bioinformatics 26: 841–842.

[8] Thorvaldsdóttir H, Robinson JT, Mesirov JP (2013) Integrative Genomics Viewer (IGV): High-performance genomics data visualization and exploration. Briefings in Bioinformatics 14: 178–192.

[9] Kaminker JS, Bergman CM, Kronmiller B, Carlson J, Svirskas R, et al. (2002) The transposable elements of the *Drosophila melanogaster* euchromatin: a genomics perspective. Genome Biology 3: RESEARCH0084.
